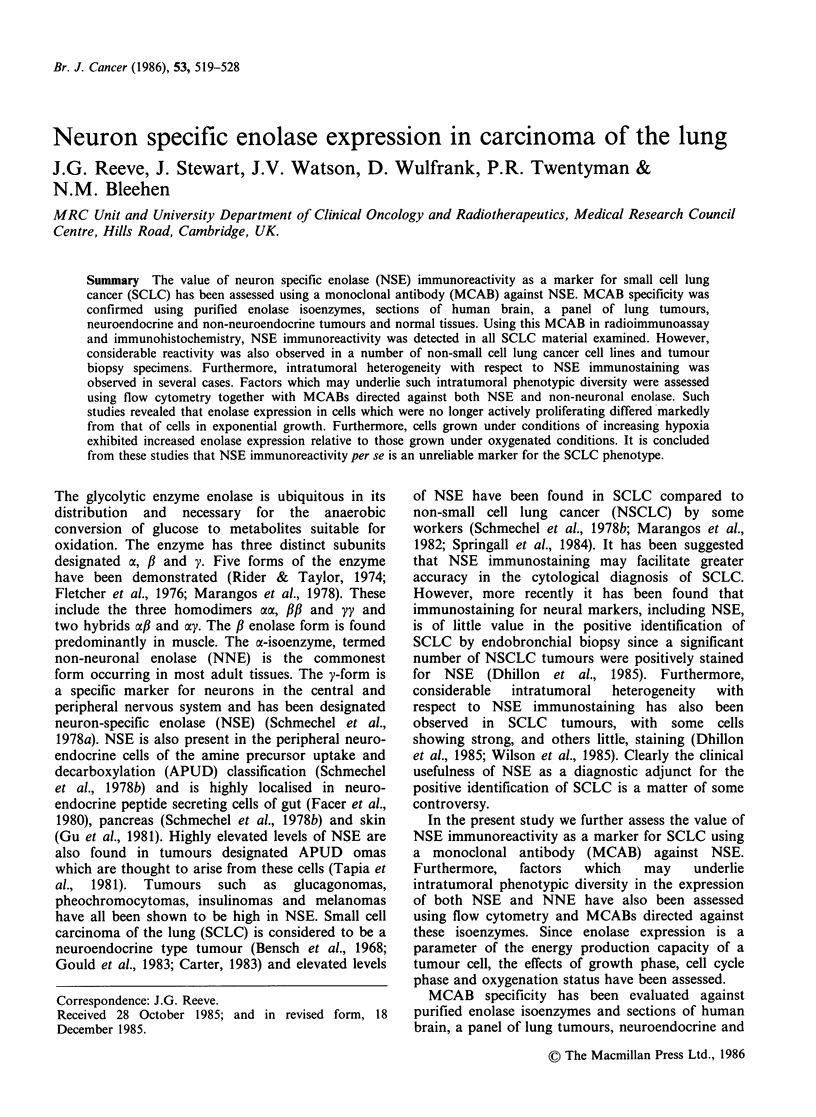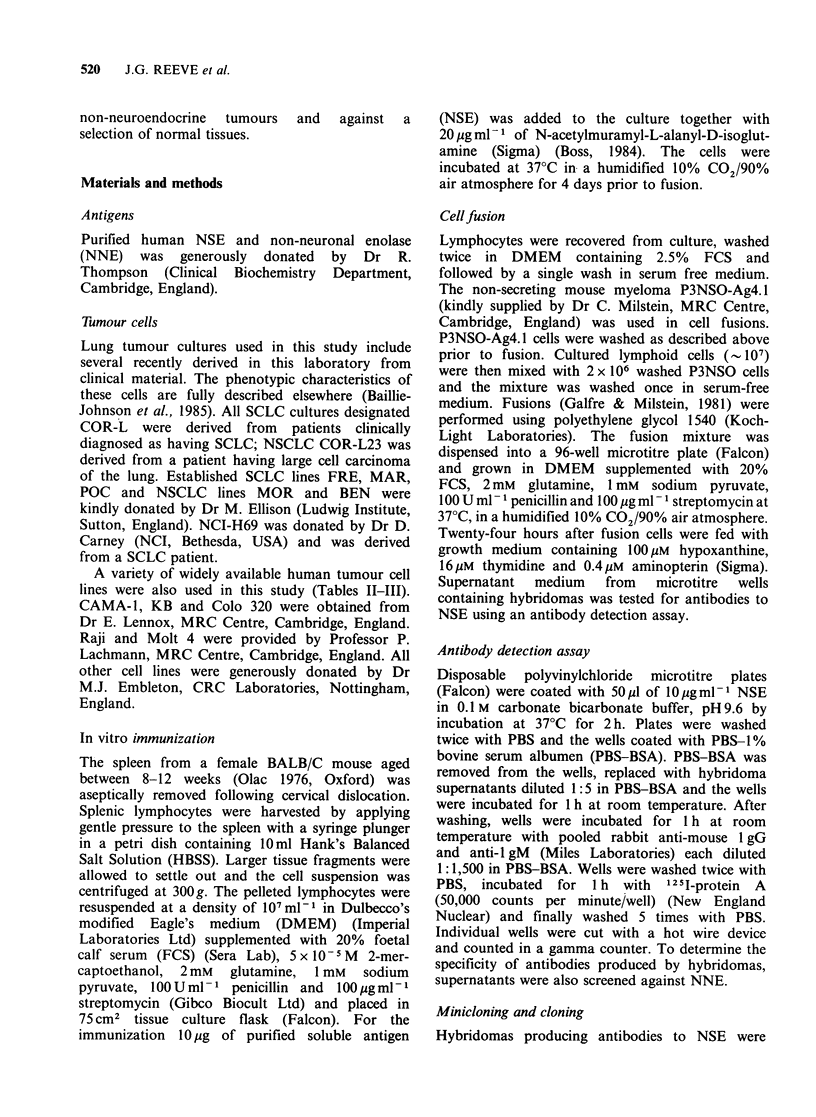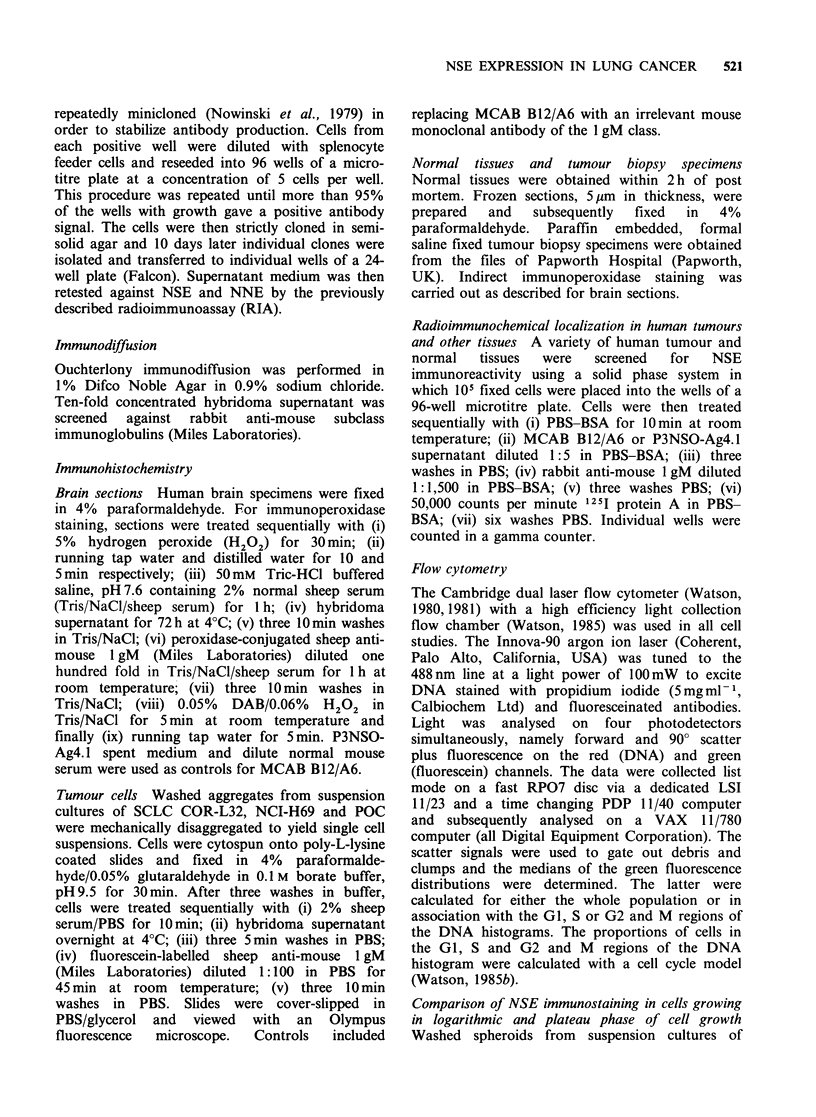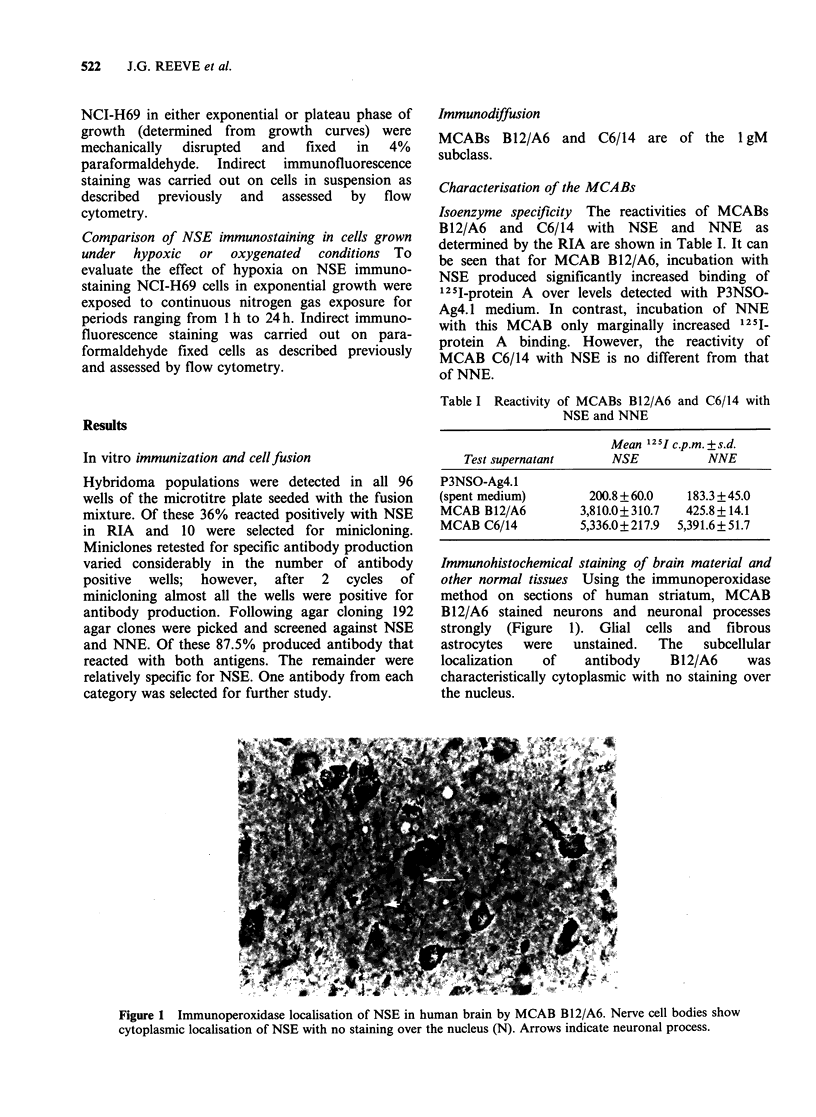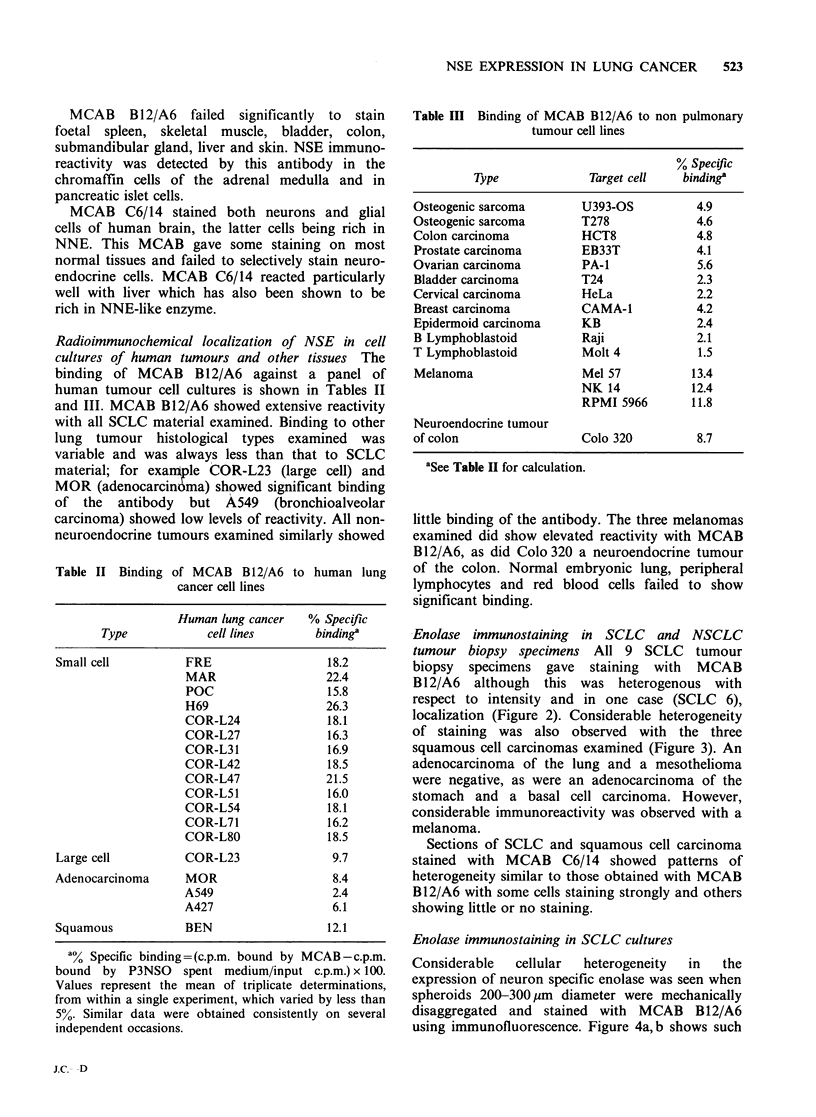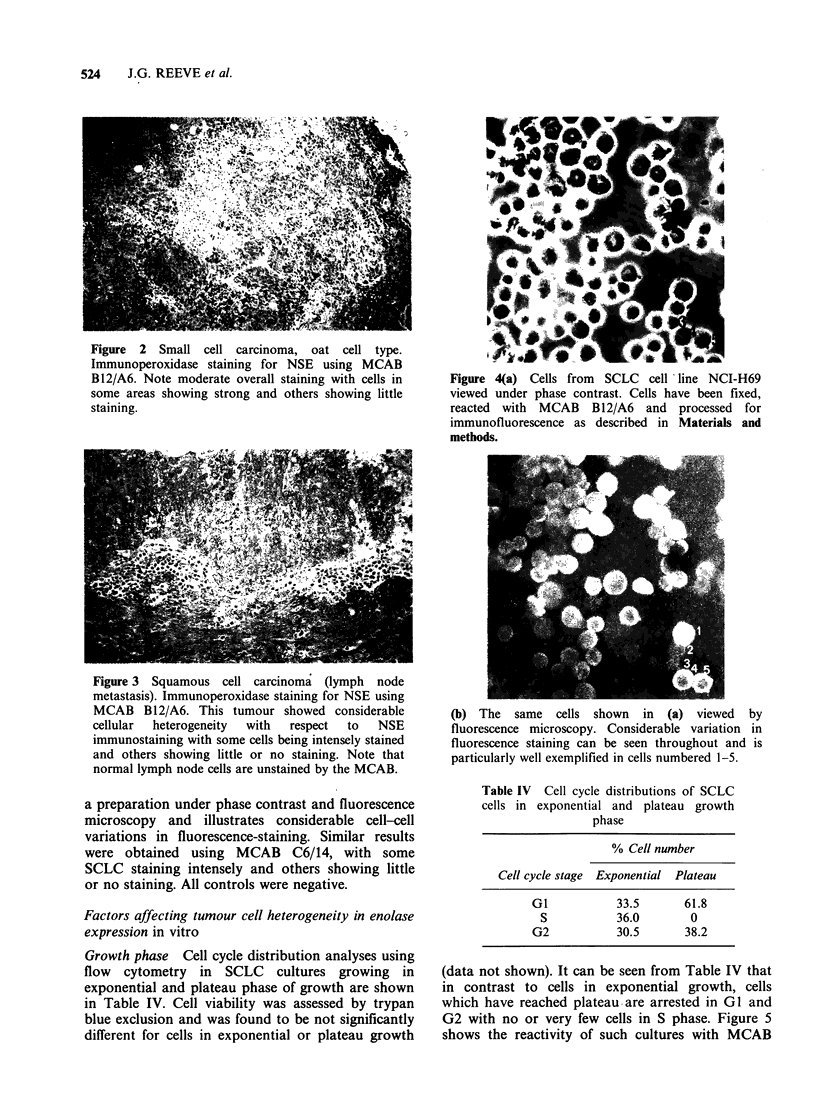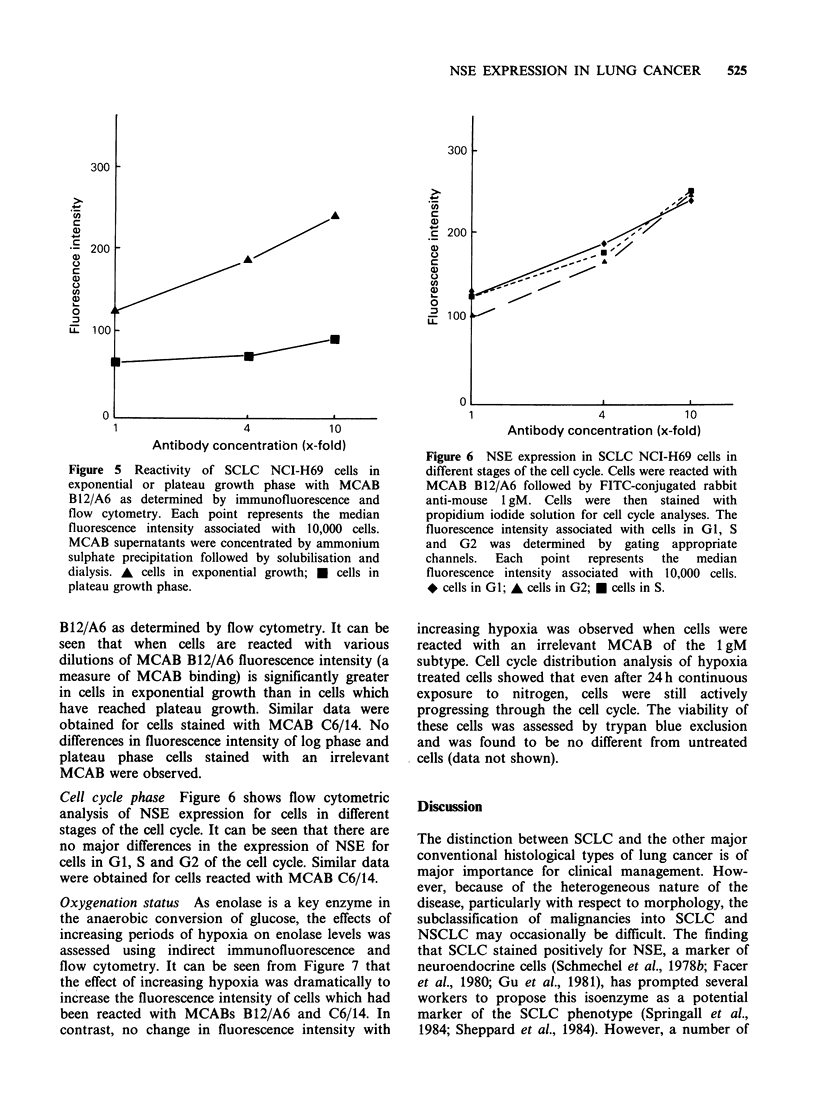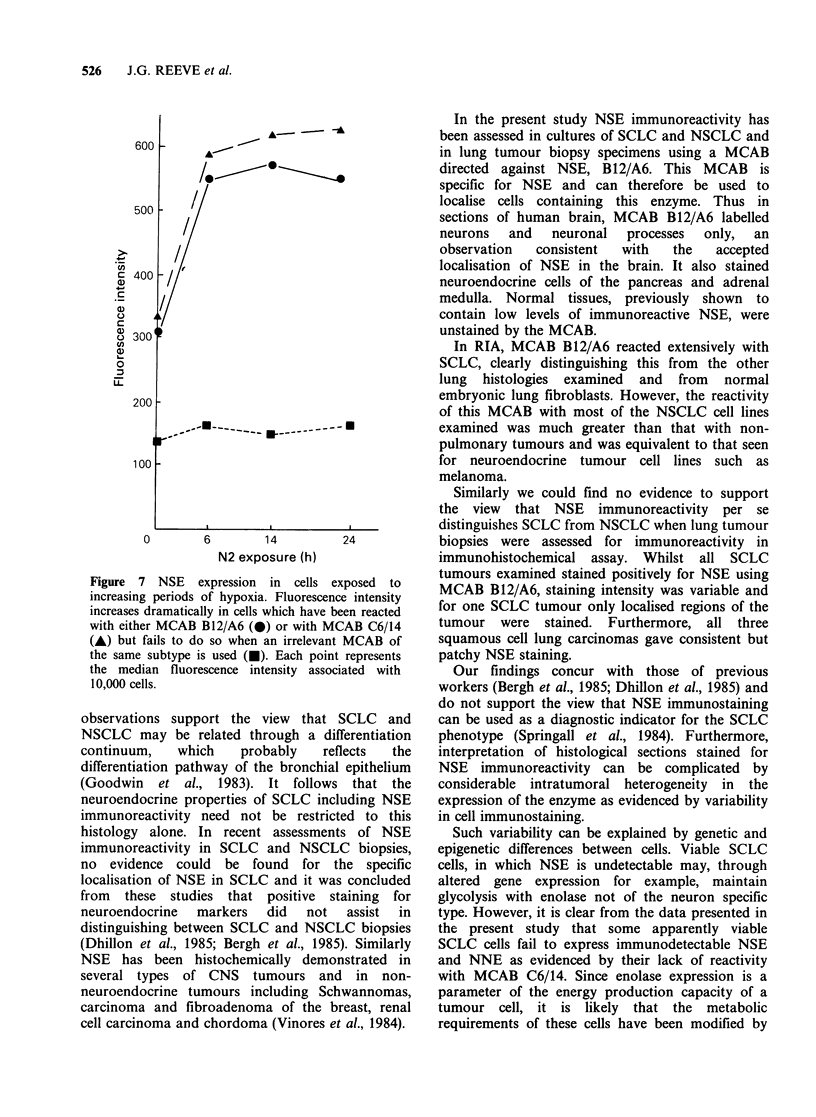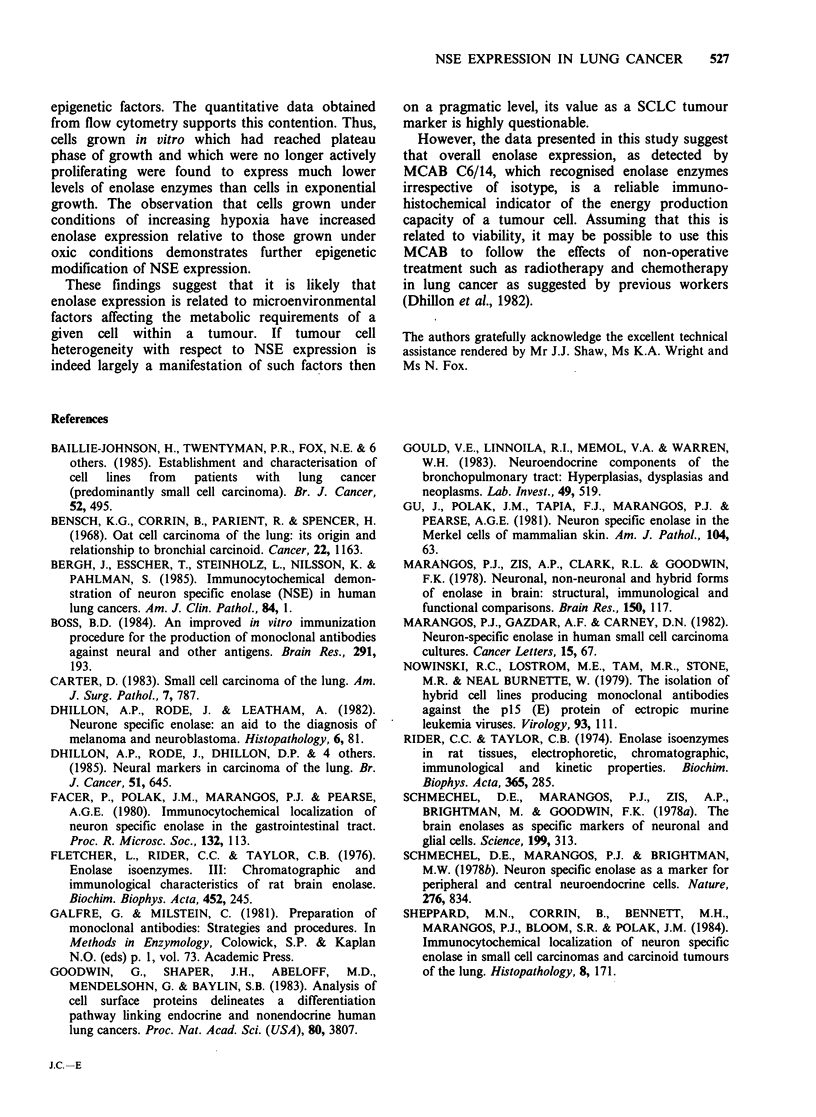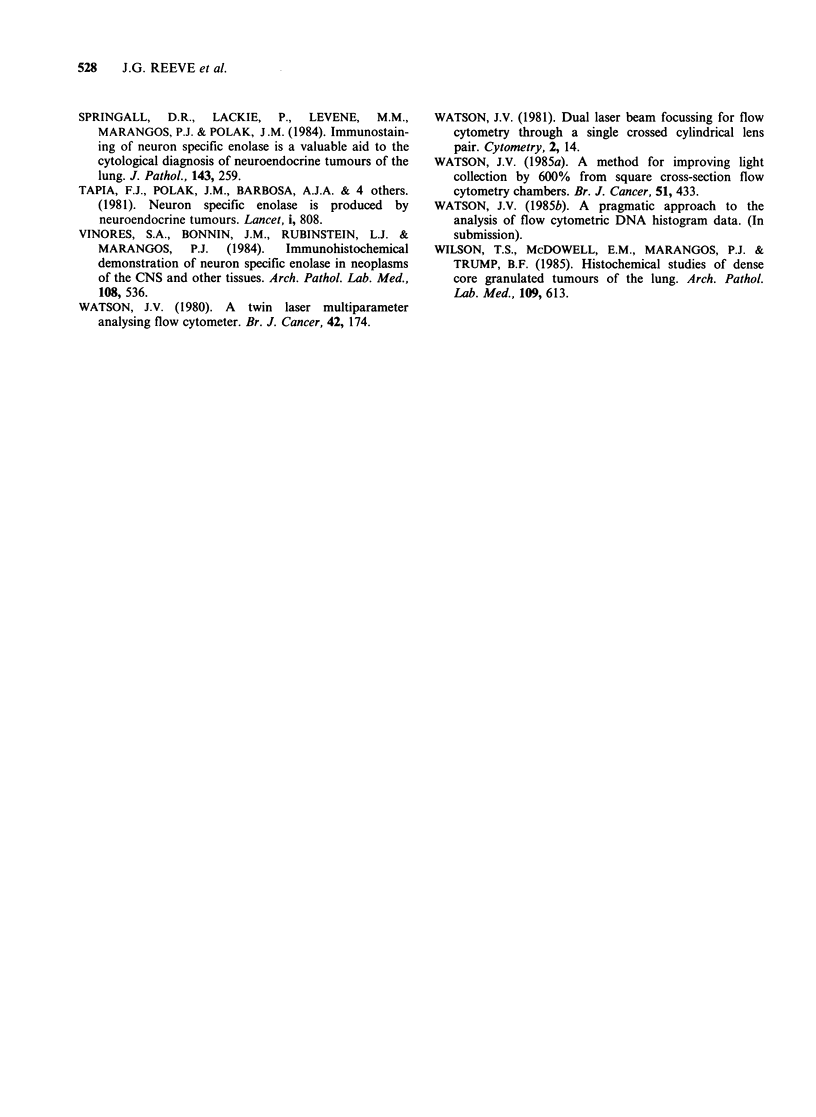# Neuron specific enolase expression in carcinoma of the lung.

**DOI:** 10.1038/bjc.1986.82

**Published:** 1986-04

**Authors:** J. G. Reeve, J. Stewart, J. V. Watson, D. Wulfrank, P. R. Twentyman, N. M. Bleehen

## Abstract

**Images:**


*Br. J. Cancer* (1986), **53**, 519–528

### Neuron specific enolase expression in carcinoma of the lung

J.G. Reeve, J. Stewart, J.V. Watson, D. Wulfrank, P.R. Twentyman & N.M. Bleehen

*MRC Unit and University Department of Clinical Oncology and Radiotherapeutics, Medical Research Council Centre, Hills Road, Cambridge, UK.*

**Summary** The value of neuron specific enolase (NSE) immunoreactivity as a marker for small cell lung cancer (SCLC) has been assessed using a monoclonal antibody (MCAB) against NSE. MCAB specificity was confirmed using purified enolase isoenzymes, sections of human brain, a panel of lung tumours, neuroendocrine and non-neuroendocrine tumours and normal tissues. Using this MCAB in radioimmunoassay and immunohistochemistry, NSE immunoreactivity was detected in all SCLC material examined. However, considerable reactivity was also observed in a number of non-small cell lung cancer cell lines and tumour biopsy specimens. Furthermore, intratumoral heterogeneity with respect to NSE immunostaining was observed in several cases. Factors which may underlie such intratumoral phenotypic diversity were assessed using flow cytometry together with MCABs directed against both NSE and non-neuronal enolase. Such studies revealed that enolase expression in cells which were no longer actively proliferating differed markedly from that of cells in exponential growth. Furthermore, cells grown under conditions of increasing hypoxia exhibited increased enolase expression relative to those grown under oxygenated conditions. It is concluded from these studies that NSE immunoreactivity *per se* is an unreliable marker for the SCLC phenotype.

The glycolytic enzyme enolase is ubiquitous in its distribution and necessary for the anaerobic conversion of glucose to metabolites suitable for oxidation. The enzyme has three distinct subunits designated $\alpha$, $\beta$ and $\gamma$. Five forms of the enzyme have been demonstrated (Rider & Taylor, 1974; Fletcher *et al.*, 1976; Marangos *et al.*, 1978). These include the three homodimers $\alpha\alpha$, $\beta\beta$ and $\gamma\gamma$ and two hybrids $\alpha\beta$ and $\alpha\gamma$. The $\beta$ enolase form is found predominantly in muscle. The $\alpha$-isoenzyme, termed non-neuronal enolase (NNE) is the commonest form occurring in most adult tissues. The $\gamma$-form is a specific marker for neurons in the central and peripheral nervous system and has been designated neuron-specific enolase (NSE) (Schmechel *et al.*, 1978*a*). NSE is also present in the peripheral neuroendocrine cells of the amine precursor uptake and decarboxylation (APUD) classification (Schmechel *et al.*, 1978*b*) and is highly localised in neuroendocrine peptide secreting cells of gut (Facer *et al.*, 1980), pancreas (Schmechel *et al.*, 1978*b*) and skin (Gu *et al.*, 1981). Highly elevated levels of NSE are also found in tumours designated APUD omas which are thought to arise from these cells (Tapia *et al.*, 1981). Tumours such as glucagonomas, pheochromocytomas, insulinomas and melanomas have all been shown to be high in NSE. Small cell carcinoma of the lung (SCLC) is considered to be a neuroendocrine type tumour (Bensch *et al.*, 1968; Gould *et al.*, 1983; Carter, 1983) and elevated levels of NSE have been found in SCLC compared to non-small cell lung cancer (NSCLC) by some workers (Schmechel *et al.*, 1978*b*; Marangos *et al.*, 1982; Springall *et al.*, 1984). It has been suggested that NSE immunostaining may facilitate greater accuracy in the cytological diagnosis of SCLC. However, more recently it has been found that immunostaining for neural markers, including NSE, is of little value in the positive identification of SCLC by endobronchial biopsy since a significant number of NSCLC tumours were positively stained for NSE (Dhillon *et al.*, 1985). Furthermore, considerable intratumoral heterogeneity with respect to NSE immunostaining has also been observed in SCLC tumours, with some cells showing strong, and others little, staining (Dhillon *et al.*, 1985; Wilson *et al.*, 1985). Clearly the clinical usefulness of NSE as a diagnostic adjunct for the positive identification of SCLC is a matter of some controversy.

In the present study we further assess the value of NSE immunoreactivity as a marker for SCLC using a monoclonal antibody (MCAB) against NSE. Furthermore, factors which may underlie intratumoral phenotypic diversity in the expression of both NSE and NNE have also been assessed using flow cytometry and MCABs directed against these isoenzymes. Since enolase expression is a parameter of the energy production capacity of a tumour cell, the effects of growth phase, cell cycle phase and oxygenation status have been assessed.

MCAB specificity has been evaluated against purified enolase isoenzymes and sections of human brain, a panel of lung tumours, neuroendocrine and

Correspondence: J.G. Reeve.
Received 28 October 1985; and in revised form, 18 December 1985.

© The Macmillan Press Ltd., 1986

non-neuroendocrine tumours and against a selection of normal tissues.

### Materials and methods

*Antigens*

Purified human NSE and non-neuronal enolase (NNE) was generously donated by Dr R. Thompson (Clinical Biochemistry Department, Cambridge, England).

*Tumour cells*

Lung tumour cultures used in this study include several recently derived in this laboratory from clinical material. The phenotypic characteristics of these cells are fully described elsewhere (Baillie-Johnson *et al.,* 1985). All SCLC cultures designated COR-L were derived from patients clinically diagnosed as having SCLC; NSCLC COR-L23 was derived from a patient having large cell carcinoma of the lung. Established SCLC lines FRE, MAR, POC and NSCLC lines MOR and BEN were kindly donated by Dr M. Ellison (Ludwig Institute, Sutton, England). NCI-H69 was donated by Dr D. Carney (NCI, Bethesda, USA) and was derived from a SCLC patient.

A variety of widely available human tumour cell lines were also used in this study (Tables II–III). CAMA-1, KB and Colo 320 were obtained from Dr E. Lennox, MRC Centre, Cambridge, England. Raji and Molt 4 were provided by Professor P. Lachmann, MRC Centre, Cambridge, England. All other cell lines were generously donated by Dr M.J. Embleton, CRC Laboratories, Nottingham, England.

In vitro *immunization*

The spleen from a female BALB/C mouse aged between 8–12 weeks (Olac 1976, Oxford) was aseptically removed following cervical dislocation. Splenic lymphocytes were harvested by applying gentle pressure to the spleen with a syringe plunger in a petri dish containing 10 ml Hank's Balanced Salt Solution (HBSS). Larger tissue fragments were allowed to settle out and the cell suspension was centrifuged at 300 *g*. The pelleted lymphocytes were resuspended at a density of $10^7\,ml^{-1}$ in Dulbecco's modified Eagle's medium (DMEM) (Imperial Laboratories Ltd) supplemented with 20% foetal calf serum (FCS) (Sera Lab), $5\times10^{-5}$ M 2-mercaptoethanol, 2 mM glutamine, 1 mM sodium pyruvate, $100\,U\,ml^{-1}$ penicillin and $100\,\mu g\,ml^{-1}$ streptomycin (Gibco Biocult Ltd) and placed in $75\,cm^2$ tissue culture flask (Falcon). For the immunization $10\,\mu g$ of purified soluble antigen (NSE) was added to the culture together with $20\,\mu g\,ml^{-1}$ of N-acetylmuramyl-L-alanyl-D-isoglutamine (Sigma) (Boss, 1984). The cells were incubated at 37°C in a humidified 10% $CO_2$/90% air atmosphere for 4 days prior to fusion.

*Cell fusion*

Lymphocytes were recovered from culture, washed twice in DMEM containing 2.5% FCS and followed by a single wash in serum free medium. The non-secreting mouse myeloma P3NSO-Ag4.1 (kindly supplied by Dr C. Milstein, MRC Centre, Cambridge, England) was used in cell fusions. P3NSO-Ag4.1 cells were washed as described above prior to fusion. Cultured lymphoid cells ($\sim10^7$) were then mixed with $2\times10^6$ washed P3NSO cells and the mixture was washed once in serum-free medium. Fusions (Galfre & Milstein, 1981) were performed using polyethylene glycol 1540 (Koch-Light Laboratories). The fusion mixture was dispensed into a 96-well microtitre plate (Falcon) and grown in DMEM supplemented with 20% FCS, 2 mM glutamine, 1 mM sodium pyruvate, $100\,U\,ml^{-1}$ penicillin and $100\,\mu g\,ml^{-1}$ streptomycin at 37°C, in a humidified 10% $CO_2$/90% air atmosphere. Twenty-four hours after fusion cells were fed with growth medium containing $100\,\mu M$ hypoxanthine, $16\,\mu M$ thymidine and $0.4\,\mu M$ aminopterin (Sigma). Supernatant medium from microtitre wells containing hybridomas was tested for antibodies to NSE using an antibody detection assay.

*Antibody detection assay*

Disposable polyvinylchloride microtitre plates (Falcon) were coated with $50\,\mu l$ of $10\,\mu g\,ml^{-1}$ NSE in 0.1 M carbonate bicarbonate buffer, pH 9.6 by incubation at 37°C for 2 h. Plates were washed twice with PBS and the wells coated with PBS–1% bovine serum albumen (PBS–BSA). PBS–BSA was removed from the wells, replaced with hybridoma supernatants diluted 1:5 in PBS–BSA and the wells were incubated for 1 h at room temperature. After washing, wells were incubated for 1 h at room temperature with pooled rabbit anti-mouse 1 gG and anti-1 gM (Miles Laboratories) each diluted 1:1,500 in PBS–BSA. Wells were washed twice with PBS, incubated for 1 h with $^{125}$I-protein A (50,000 counts per minute/well) (New England Nuclear) and finally washed 5 times with PBS. Individual wells were cut with a hot wire device and counted in a gamma counter. To determine the specificity of antibodies produced by hybridomas, supernatants were also screened against NNE.

*Minicloning and cloning*

Hybridomas producing antibodies to NSE were repeatedly minicloned (Nowinski *et al.*, 1979) in order to stabilize antibody production. Cells from each positive well were diluted with splenocyte feeder cells and reseeded into 96 wells of a microtitre plate at a concentration of 5 cells per well. This procedure was repeated until more than 95% of the wells with growth gave a positive antibody signal. The cells were then strictly cloned in semisolid agar and 10 days later individual clones were isolated and transferred to individual wells of a 24-well plate (Falcon). Supernatant medium was then retested against NSE and NNE by the previously described radioimmunoassay (RIA).

*Immunodiffusion*

Ouchterlony immunodiffusion was performed in 1% Difco Noble Agar in 0.9% sodium chloride. Ten-fold concentrated hybridoma supernatant was screened against rabbit anti-mouse subclass immunoglobulins (Miles Laboratories).

*Immunohistochemistry*

*Brain sections* Human brain specimens were fixed in 4% paraformaldehyde. For immunoperoxidase staining, sections were treated sequentially with (i) 5% hydrogen peroxide ($H_2O_2$) for 30min; (ii) running tap water and distilled water for 10 and 5min respectively; (iii) 50mM Tric-HCl buffered saline, pH7.6 containing 2% normal sheep serum (Tris/NaCl/sheep serum) for 1h; (iv) hybridoma supernatant for 72h at 4°C; (v) three 10min washes in Tris/NaCl; (vi) peroxidase-conjugated sheep anti-mouse 1gM (Miles Laboratories) diluted one hundred fold in Tris/NaCl/sheep serum for 1h at room temperature; (vii) three 10min washes in Tris/NaCl; (viii) 0.05% DAB/0.06% $H_2O_2$ in Tris/NaCl for 5min at room temperature and finally (ix) running tap water for 5min. P3NSO-Ag4.1 spent medium and dilute normal mouse serum were used as controls for MCAB B12/A6.

*Tumour cells* Washed aggregates from suspension cultures of SCLC COR-L32, NCI-H69 and POC were mechanically disaggregated to yield single cell suspensions. Cells were cytospun onto poly-L-lysine coated slides and fixed in 4% paraformaldehyde/0.05% glutaraldehyde in 0.1M borate buffer, pH9.5 for 30min. After three washes in buffer, cells were treated sequentially with (i) 2% sheep serum/PBS for 10min; (ii) hybridoma supernatant overnight at 4°C; (iii) three 5min washes in PBS; (iv) fluorescein-labelled sheep anti-mouse 1gM (Miles Laboratories) diluted 1:100 in PBS for 45min at room temperature; (v) three 10min washes in PBS. Slides were cover-slipped in PBS/glycerol and viewed with an Olympus fluorescence microscope. Controls included replacing MCAB B12/A6 with an irrelevant mouse monoclonal antibody of the 1gM class.

*Normal tissues and tumour biopsy specimens* Normal tissues were obtained within 2h of post mortem. Frozen sections, 5 μm in thickness, were prepared and subsequently fixed in 4% paraformaldehyde. Paraffin embedded, formal saline fixed tumour biopsy specimens were obtained from the files of Papworth Hospital (Papworth, UK). Indirect immunoperoxidase staining was carried out as described for brain sections.

*Radioimmunochemical localization in human tumours and other tissues* A variety of human tumour and normal tissues were screened for NSE immunoreactivity using a solid phase system in which $10^5$ fixed cells were placed into the wells of a 96-well microtitre plate. Cells were then treated sequentially with (i) PBS–BSA for 10min at room temperature; (ii) MCAB B12/A6 or P3NSO-Ag4.1 supernatant diluted 1:5 in PBS–BSA; (iii) three washes in PBS; (iv) rabbit anti-mouse 1gM diluted 1:1,500 in PBS–BSA; (v) three washes PBS; (vi) 50,000 counts per minute $^{125}$I protein A in PBS–BSA; (vii) six washes PBS. Individual wells were counted in a gamma counter.

*Flow cytometry*

The Cambridge dual laser flow cytometer (Watson, 1980, 1981) with a high efficiency light collection flow chamber (Watson, 1985) was used in all cell studies. The Innova-90 argon ion laser (Coherent, Palo Alto, California, USA) was tuned to the 488nm line at a light power of 100mW to excite DNA stained with propidium iodide (5mgml$^{-1}$, Calbiochem Ltd) and fluoresceinated antibodies. Light was analysed on four photodetectors simultaneously, namely forward and 90° scatter plus fluorescence on the red (DNA) and green (fluorescein) channels. The data were collected list mode on a fast RPO7 disc via a dedicated LSI 11/23 and a time changing PDP 11/40 computer and subsequently analysed on a VAX 11/780 computer (all Digital Equipment Corporation). The scatter signals were used to gate out debris and clumps and the medians of the green fluorescence distributions were determined. The latter were calculated for either the whole population or in association with the G1, S or G2 and M regions of the DNA histograms. The proportions of cells in the G1, S and G2 and M regions of the DNA histogram were calculated with a cell cycle model (Watson, 1985*b*).

*Comparison of NSE immunostaining in cells growing in logarithmic and plateau phase of cell growth* Washed spheroids from suspension cultures of

NCI-H69 in either exponential or plateau phase of growth (determined from growth curves) were mechanically disrupted and fixed in 4% paraformaldehyde. Indirect immunofluorescence staining was carried out on cells in suspension as described previously and assessed by flow cytometry.

*Comparison of NSE immunostaining in cells grown under hypoxic or oxygenated conditions* To evaluate the effect of hypoxia on NSE immunostaining NCI-H69 cells in exponential growth were exposed to continuous nitrogen gas exposure for periods ranging from 1 h to 24 h. Indirect immunofluorescence staining was carried out on paraformaldehyde fixed cells as described previously and assessed by flow cytometry.

### Results

### In vitro *immunization and cell fusion*

Hybridoma populations were detected in all 96 wells of the microtitre plate seeded with the fusion mixture. Of these 36% reacted positively with NSE in RIA and 10 were selected for minicloning. Miniclones retested for specific antibody production varied considerably in the number of antibody positive wells; however, after 2 cycles of minicloning almost all the wells were positive for antibody production. Following agar cloning 192 agar clones were picked and screened against NSE and NNE. Of these 87.5% produced antibody that reacted with both antigens. The remainder were relatively specific for NSE. One antibody from each category was selected for further study.

### *Immunodiffusion*

MCABs B12/A6 and C6/14 are of the 1gM subclass.

### *Characterisation of the MCABs*

*Isoenzyme specificity* The reactivities of MCABs B12/A6 and C6/14 with NSE and NNE as determined by the RIA are shown in Table I. It can be seen that for MCAB B12/A6, incubation with NSE produced significantly increased binding of $^{125}$I-protein A over levels detected with P3NSO-Ag4.1 medium. In contrast, incubation of NNE with this MCAB only marginally increased $^{125}$I-protein A binding. However, the reactivity of MCAB C6/14 with NSE is no different from that of NNE.

Table I Reactivity of MCABs B12/A6 and C6/14 with NSE and NNE

| *Test supernatant* | *Mean $^{125}I$ c.p.m. ± s.d.* NSE | NNE |
|---|---|---|
| P3NSO-Ag4.1 (spent medium) | 200.8 ± 60.0 | 183.3 ± 45.0 |
| MCAB B12/A6 | 3,810.0 ± 310.7 | 425.8 ± 14.1 |
| MCAB C6/14 | 5,336.0 ± 217.9 | 5,391.6 ± 51.7 |

*Immunohistochemical staining of brain material and other normal tissues* Using the immunoperoxidase method on sections of human striatum, MCAB B12/A6 stained neurons and neuronal processes strongly (Figure 1). Glial cells and fibrous astrocytes were unstained. The subcellular localization of antibody B12/A6 was characteristically cytoplasmic with no staining over the nucleus.

**Figure 1** Immunoperoxidase localisation of NSE in human brain by MCAB B12/A6. Nerve cell bodies show cytoplasmic localisation of NSE with no staining over the nucleus (N). Arrows indicate neuronal process.

MCAB B12/A6 failed significantly to stain foetal spleen, skeletal muscle, bladder, colon, submandibular gland, liver and skin. NSE immunoreactivity was detected by this antibody in the chromaffin cells of the adrenal medulla and in pancreatic islet cells.

MCAB C6/14 stained both neurons and glial cells of human brain, the latter cells being rich in NNE. This MCAB gave some staining on most normal tissues and failed to selectively stain neuroendocrine cells. MCAB C6/14 reacted particularly well with liver which has also been shown to be rich in NNE-like enzyme.

*Radioimmunochemical localization of NSE in cell cultures of human tumours and other tissues* The binding of MCAB B12/A6 against a panel of human tumour cell cultures is shown in Tables II and III. MCAB B12/A6 showed extensive reactivity with all SCLC material examined. Binding to other lung tumour histological types examined was variable and was always less than that to SCLC material; for example COR-L23 (large cell) and MOR (adenocarcinoma) showed significant binding of the antibody but A549 (bronchioalveolar carcinoma) showed low levels of reactivity. All non-neuroendocrine tumours examined similarly showed little binding of the antibody. The three melanomas examined did show elevated reactivity with MCAB B12/A6, as did Colo 320 a neuroendocrine tumour of the colon. Normal embryonic lung, peripheral lymphocytes and red blood cells failed to show significant binding.

**Table II** Binding of MCAB B12/A6 to human lung cancer cell lines

| *Type* | *Human lung cancer cell lines* | *% Specific binding*[a] |
|---|---|---|
| Small cell | FRE | 18.2 |
| | MAR | 22.4 |
| | POC | 15.8 |
| | H69 | 26.3 |
| | COR-L24 | 18.1 |
| | COR-L27 | 16.3 |
| | COR-L31 | 16.9 |
| | COR-L42 | 18.5 |
| | COR-L47 | 21.5 |
| | COR-L51 | 16.0 |
| | COR-L54 | 18.1 |
| | COR-L71 | 16.2 |
| | COR-L80 | 18.5 |
| Large cell | COR-L23 | 9.7 |
| Adenocarcinoma | MOR | 8.4 |
| | A549 | 2.4 |
| | A427 | 6.1 |
| Squamous | BEN | 12.1 |

[a]% Specific binding = (c.p.m. bound by MCAB − c.p.m. bound by P3NSO spent medium/input c.p.m.) × 100. Values represent the mean of triplicate determinations, from within a single experiment, which varied by less than 5%. Similar data were obtained consistently on several independent occasions.

**Table III** Binding of MCAB B12/A6 to non pulmonary tumour cell lines

| *Type* | *Target cell* | *% Specific binding*[a] |
|---|---|---|
| Osteogenic sarcoma | U393-OS | 4.9 |
| Osteogenic sarcoma | T278 | 4.6 |
| Colon carcinoma | HCT8 | 4.8 |
| Prostate carcinoma | EB33T | 4.1 |
| Ovarian carcinoma | PA-1 | 5.6 |
| Bladder carcinoma | T24 | 2.3 |
| Cervical carcinoma | HeLa | 2.2 |
| Breast carcinoma | CAMA-1 | 4.2 |
| Epidermoid carcinoma | KB | 2.4 |
| B Lymphoblastoid | Raji | 2.1 |
| T Lymphoblastoid | Molt 4 | 1.5 |
| Melanoma | Mel 57 | 13.4 |
| | NK 14 | 12.4 |
| | RPMI 5966 | 11.8 |
| Neuroendocrine tumour of colon | Colo 320 | 8.7 |

[a]See **Table II** for calculation.

*Enolase immunostaining in SCLC and NSCLC tumour biopsy specimens* All 9 SCLC tumour biopsy specimens gave staining with MCAB B12/A6 although this was heterogenous with respect to intensity and in one case (SCLC 6), localization (Figure 2). Considerable heterogeneity of staining was also observed with the three squamous cell carcinomas examined (Figure 3). An adenocarcinoma of the lung and a mesothelioma were negative, as were an adenocarcinoma of the stomach and a basal cell carcinoma. However, considerable immunoreactivity was observed with a melanoma.

Sections of SCLC and squamous cell carcinoma stained with MCAB C6/14 showed patterns of heterogeneity similar to those obtained with MCAB B12/A6 with some cells staining strongly and others showing little or no staining.

*Enolase immunostaining in SCLC cultures*

Considerable cellular heterogeneity in the expression of neuron specific enolase was seen when spheroids 200–300 $\mu$m diameter were mechanically disaggregated and stained with MCAB B12/A6 using immunofluorescence. Figure 4a, b shows such

**Figure 2** Small cell carcinoma, oat cell type. Immunoperoxidase staining for NSE using MCAB B12/A6. Note moderate overall staining with cells in some areas showing strong and others showing little staining.

**Figure 3** Squamous cell carcinoma (lymph node metastasis). Immunoperoxidase staining for NSE using MCAB B12/A6. This tumour showed considerable cellular heterogeneity with respect to NSE immunostaining with some cells being intensely stained and others showing little or no staining. Note that normal lymph node cells are unstained by the MCAB.

a preparation under phase contrast and fluorescence microscopy and illustrates considerable cell–cell variations in fluorescence-staining. Similar results were obtained using MCAB C6/14, with some SCLC staining intensely and others showing little or no staining. All controls were negative.

*Factors affecting tumour cell heterogeneity in enolase expression* in vitro

*Growth phase* Cell cycle distribution analyses using flow cytometry in SCLC cultures growing in exponential and plateau phase of growth are shown in Table IV. Cell viability was assessed by trypan blue exclusion and was found to be not significantly different for cells in exponential or plateau growth

**Figure 4(a)** Cells from SCLC cell line NCI-H69 viewed under phase contrast. Cells have been fixed, reacted with MCAB B12/A6 and processed for immunofluorescence as described in **Materials and methods.**

**(b)** The same cells shown in **(a)** viewed by fluorescence microscopy. Considerable variation in fluorescence staining can be seen throughout and is particularly well exemplified in cells numbered 1–5.

**Table IV** Cell cycle distributions of SCLC cells in exponential and plateau growth phase

| | *% Cell number* | |
|---|---|---|
| *Cell cycle stage* | *Exponential* | *Plateau* |
| G1 | 33.5 | 61.8 |
| S | 36.0 | 0 |
| G2 | 30.5 | 38.2 |

(data not shown). It can be seen from Table IV that in contrast to cells in exponential growth, cells which have reached plateau are arrested in G1 and G2 with no or very few cells in S phase. Figure 5 shows the reactivity of such cultures with MCAB

**Figure 5** Reactivity of SCLC NCI-H69 cells in exponential or plateau growth phase with MCAB B12/A6 as determined by immunofluorescence and flow cytometry. Each point represents the median fluorescence intensity associated with 10,000 cells. MCAB supernatants were concentrated by ammonium sulphate precipitation followed by solubilisation and dialysis. ▲ cells in exponential growth; ■ cells in plateau growth phase.

**Figure 6** NSE expression in SCLC NCI-H69 cells in different stages of the cell cycle. Cells were reacted with MCAB B12/A6 followed by FITC-conjugated rabbit anti-mouse 1 gM. Cells were then stained with propidium iodide solution for cell cycle analyses. The fluorescence intensity associated with cells in G1, S and G2 was determined by gating appropriate channels. Each point represents the median fluorescence intensity associated with 10,000 cells. ◆ cells in G1; ▲ cells in G2; ■ cells in S.

B12/A6 as determined by flow cytometry. It can be seen that when cells are reacted with various dilutions of MCAB B12/A6 fluorescence intensity (a measure of MCAB binding) is significantly greater in cells in exponential growth than in cells which have reached plateau growth. Similar data were obtained for cells stained with MCAB C6/14. No differences in fluorescence intensity of log phase and plateau phase cells stained with an irrelevant MCAB were observed.

*Cell cycle phase* Figure 6 shows flow cytometric analysis of NSE expression for cells in different stages of the cell cycle. It can be seen that there are no major differences in the expression of NSE for cells in G1, S and G2 of the cell cycle. Similar data were obtained for cells reacted with MCAB C6/14.

*Oxygenation status* As enolase is a key enzyme in the anaerobic conversion of glucose, the effects of increasing periods of hypoxia on enolase levels was assessed using indirect immunofluorescence and flow cytometry. It can be seen from Figure 7 that the effect of increasing hypoxia was dramatically to increase the fluorescence intensity of cells which had been reacted with MCABs B12/A6 and C6/14. In contrast, no change in fluorescence intensity with increasing hypoxia was observed when cells were reacted with an irrelevant MCAB of the 1 gM subtype. Cell cycle distribution analysis of hypoxia treated cells showed that even after 24 h continuous exposure to nitrogen, cells were still actively progressing through the cell cycle. The viability of these cells was assessed by trypan blue exclusion and was found to be no different from untreated cells (data not shown).

### Discussion

The distinction between SCLC and the other major conventional histological types of lung cancer is of major importance for clinical management. However, because of the heterogeneous nature of the disease, particularly with respect to morphology, the subclassification of malignancies into SCLC and NSCLC may occasionally be difficult. The finding that SCLC stained positively for NSE, a marker of neuroendocrine cells (Schmechel *et al.*, 1978*b*; Facer *et al.*, 1980; Gu *et al.*, 1981), has prompted several workers to propose this isoenzyme as a potential marker of the SCLC phenotype (Springall *et al.*, 1984; Sheppard *et al.*, 1984). However, a number of

**Figure 7** NSE expression in cells exposed to increasing periods of hypoxia. Fluorescence intensity increases dramatically in cells which have been reacted with either MCAB B12/A6 (●) or with MCAB C6/14 (▲) but fails to do so when an irrelevant MCAB of the same subtype is used (■). Each point represents the median fluorescence intensity associated with 10,000 cells.

observations support the view that SCLC and NSCLC may be related through a differentiation continuum, which probably reflects the differentiation pathway of the bronchial epithelium (Goodwin *et al.*, 1983). It follows that the neuroendocrine properties of SCLC including NSE immunoreactivity need not be restricted to this histology alone. In recent assessments of NSE immunoreactivity in SCLC and NSCLC biopsies, no evidence could be found for the specific localisation of NSE in SCLC and it was concluded from these studies that positive staining for neuroendocrine markers did not assist in distinguishing between SCLC and NSCLC biopsies (Dhillon *et al.*, 1985; Bergh *et al.*, 1985). Similarly NSE has been histochemically demonstrated in several types of CNS tumours and in non-neuroendocrine tumours including Schwannomas, carcinoma and fibroadenoma of the breast, renal cell carcinoma and chordoma (Vinores *et al.*, 1984).

In the present study NSE immunoreactivity has been assessed in cultures of SCLC and NSCLC and in lung tumour biopsy specimens using a MCAB directed against NSE, B12/A6. This MCAB is specific for NSE and can therefore be used to localise cells containing this enzyme. Thus in sections of human brain, MCAB B12/A6 labelled neurons and neuronal processes only, an observation consistent with the accepted localisation of NSE in the brain. It also stained neuroendocrine cells of the pancreas and adrenal medulla. Normal tissues, previously shown to contain low levels of immunoreactive NSE, were unstained by the MCAB.

In RIA, MCAB B12/A6 reacted extensively with SCLC, clearly distinguishing this from the other lung histologies examined and from normal embryonic lung fibroblasts. However, the reactivity of this MCAB with most of the NSCLC cell lines examined was much greater than that with non-pulmonary tumours and was equivalent to that seen for neuroendocrine tumour cell lines such as melanoma.

Similarly we could find no evidence to support the view that NSE immunoreactivity per se distinguishes SCLC from NSCLC when lung tumour biopsies were assessed for immunoreactivity in immunohistochemical assay. Whilst all SCLC tumours examined stained positively for NSE using MCAB B12/A6, staining intensity was variable and for one SCLC tumour only localised regions of the tumour were stained. Furthermore, all three squamous cell lung carcinomas gave consistent but patchy NSE staining.

Our findings concur with those of previous workers (Bergh *et al.*, 1985; Dhillon *et al.*, 1985) and do not support the view that NSE immunostaining can be used as a diagnostic indicator for the SCLC phenotype (Springall *et al.*, 1984). Furthermore, interpretation of histological sections stained for NSE immunoreactivity can be complicated by considerable intratumoral heterogeneity in the expression of the enzyme as evidenced by variability in cell immunostaining.

Such variability can be explained by genetic and epigenetic differences between cells. Viable SCLC cells, in which NSE is undetectable may, through altered gene expression for example, maintain glycolysis with enolase not of the neuron specific type. However, it is clear from the data presented in the present study that some apparently viable SCLC cells fail to express immunodetectable NSE and NNE as evidenced by their lack of reactivity with MCAB C6/14. Since enolase expression is a parameter of the energy production capacity of a tumour cell, it is likely that the metabolic requirements of these cells have been modified by epigenetic factors. The quantitative data obtained from flow cytometry supports this contention. Thus, cells grown *in vitro* which had reached plateau phase of growth and which were no longer actively proliferating were found to express much lower levels of enolase enzymes than cells in exponential growth. The observation that cells grown under conditions of increasing hypoxia have increased enolase expression relative to those grown under oxic conditions demonstrates further epigenetic modification of NSE expression.

These findings suggest that it is likely that enolase expression is related to microenvironmental factors affecting the metabolic requirements of a given cell within a tumour. If tumour cell heterogeneity with respect to NSE expression is indeed largely a manifestation of such factors then on a pragmatic level, its value as a SCLC tumour marker is highly questionable.

However, the data presented in this study suggest that overall enolase expression, as detected by MCAB C6/14, which recognised enolase enzymes irrespective of isotype, is a reliable immunohistochemical indicator of the energy production capacity of a tumour cell. Assuming that this is related to viability, it may be possible to use this MCAB to follow the effects of non-operative treatment such as radiotherapy and chemotherapy in lung cancer as suggested by previous workers (Dhillon *et al.*, 1982).

The authors gratefully acknowledge the excellent technical assistance rendered by Mr J.J. Shaw, Ms K.A. Wright and Ms N. Fox.

### References

BAILLIE-JOHNSON, H., TWENTYMAN, P.R., FOX, N.E. & 6 others. (1985). Establishment and characterisation of cell lines from patients with lung cancer (predominantly small cell carcinoma). *Br. J. Cancer*, **52**, 495.

BENSCH, K.G., CORRIN, B., PARIENT, R. & SPENCER, H. (1968). Oat cell carcinoma of the lung: its origin and relationship to bronchial carcinoid. *Cancer*, **22**, 1163.

BERGH, J., ESSCHER, T., STEINHOLZ, L., NILSSON, K. & PAHLMAN, S. (1985). Immunocytochemical demonstration of neuron specific enolase (NSE) in human lung cancers. *Am. J. Clin. Pathol.*, **84**, 1.

BOSS, B.D. (1984). An improved *in vitro* immunization procedure for the production of monoclonal antibodies against neural and other antigens. *Brain Res.*, **291**, 193.

CARTER, D. (1983). Small cell carcinoma of the lung. *Am. J. Surg. Pathol.*, **7**, 787.

DHILLON, A.P., RODE, J. & LEATHAM, A. (1982). Neurone specific enolase: an aid to the diagnosis of melanoma and neuroblastoma. *Histopathology*, **6**, 81.

DHILLON, A.P., RODE, J., DHILLON, D.P. & 4 others. (1985). Neural markers in carcinoma of the lung. *Br. J. Cancer*, **51**, 645.

FACER, P., POLAK, J.M., MARANGOS, P.J. & PEARSE, A.G.E. (1980). Immunocytochemical localization of neuron specific enolase in the gastrointestinal tract. *Proc. R. Microsc. Soc.*, **132**, 113.

FLETCHER, L., RIDER, C.C. & TAYLOR, C.B. (1976). Enolase isoenzymes. III: Chromatographic and immunological characteristics of rat brain enolase. *Biochim. Biophys. Acta*, **452**, 245.

GALFRE, G. & MILSTEIN, C. (1981). Preparation of monoclonal antibodies: Strategies and procedures. In *Methods in Enzymology*, Colowick, S.P. & Kaplan N.O. (eds) p. 1, vol. 73. Academic Press.

GOODWIN, G., SHAPER, J.H., ABELOFF, M.D., MENDELSOHN, G. & BAYLIN, S.B. (1983). Analysis of cell surface proteins delineates a differentiation pathway linking endocrine and nonendocrine human lung cancers. *Proc. Nat. Acad. Sci. (USA)*, **80**, 3807.

GOULD, V.E., LINNOILA, R.I., MEMOL, V.A. & WARREN, W.H. (1983). Neuroendocrine components of the bronchopulmonary tract: Hyperplasias, dysplasias and neoplasms. *Lab. Invest.*, **49**, 519.

GU, J., POLAK, J.M., TAPIA, F.J., MARANGOS, P.J. & PEARSE, A.G.E. (1981). Neuron specific enolase in the Merkel cells of mammalian skin. *Am. J. Pathol.*, **104**, 63.

MARANGOS, P.J., ZIS, A.P., CLARK, R.L. & GOODWIN, F.K. (1978). Neuronal, non-neuronal and hybrid forms of enolase in brain: structural, immunological and functional comparisons. *Brain Res.*, **150**, 117.

MARANGOS, P.J., GAZDAR, A.F. & CARNEY, D.N. (1982). Neuron-specific enolase in human small cell carcinoma cultures. *Cancer Letters*, **15**, 67.

NOWINSKI, R.C., LOSTROM, M.E., TAM, M.R., STONE, M.R. & NEAL BURNETTE, W. (1979). The isolation of hybrid cell lines producing monoclonal antibodies against the p15 (E) protein of ectropic murine leukemia viruses. *Virology*, **93**, 111.

RIDER, C.C. & TAYLOR, C.B. (1974). Enolase isoenzymes in rat tissues, electrophoretic, chromatographic, immunological and kinetic properties. *Biochim. Biophys. Acta*, **365**, 285.

SCHMECHEL, D.E., MARANGOS, P.J., ZIS, A.P., BRIGHTMAN, M. & GOODWIN, F.K. (1978*a*). The brain enolases as specific markers of neuronal and glial cells. *Science*, **199**, 313.

SCHMECHEL, D.E., MARANGOS, P.J. & BRIGHTMAN, M.W. (1978*b*). Neuron specific enolase as a marker for peripheral and central neuroendocrine cells. *Nature*, **276**, 834.

SHEPPARD, M.N., CORRIN, B., BENNETT, M.H., MARANGOS, P.J., BLOOM, S.R. & POLAK, J.M. (1984). Immunocytochemical localization of neuron specific enolase in small cell carcinomas and carcinoid tumours of the lung. *Histopathology*, **8**, 171.

SPRINGALL, D.R., LACKIE, P., LEVENE, M.M., MARANGOS, P.J. & POLAK, J.M. (1984). Immunostaining of neuron specific enolase is a valuable aid to the cytological diagnosis of neuroendocrine tumours of the lung. *J. Pathol.*, **143**, 259.

TAPIA, F.J., POLAK, J.M., BARBOSA, A.J.A. & 4 others. (1981). Neuron specific enolase is produced by neuroendocrine tumours. *Lancet*, **i**, 808.

VINORES, S.A., BONNIN, J.M., RUBINSTEIN, L.J. & MARANGOS, P.J. (1984). Immunohistochemical demonstration of neuron specific enolase in neoplasms of the CNS and other tissues. *Arch. Pathol. Lab. Med.*, **108**, 536.

WATSON, J.V. (1980). A twin laser multiparameter analysing flow cytometer. *Br. J. Cancer*, **42**, 174.

WATSON, J.V. (1981). Dual laser beam focussing for flow cytometry through a single crossed cylindrical lens pair. *Cytometry*, **2**, 14.

WATSON, J.V. (1985*a*). A method for improving light collection by 600% from square cross-section flow cytometry chambers. *Br. J. Cancer*, **51**, 433.

WATSON, J.V. (1985*b*). A pragmatic approach to the analysis of flow cytometric DNA histogram data. (In submission).

WILSON, T.S., McDOWELL, E.M., MARANGOS, P.J. & TRUMP, B.F. (1985). Histochemical studies of dense core granulated tumours of the lung. *Arch. Pathol. Lab. Med.*, **109**, 613.